# Increased Osteogenic Potential of Pre-Osteoblasts on Three-Dimensional Printed Scaffolds Compared to Porous Scaffolds for Bone Regeneration

**DOI:** 10.29252/ibj.25.2.78

**Published:** 2021-01-13

**Authors:** Yasaman Zamani, Ghassem Amoabediny, Javad Mohammadi, Behrouz Zandieh-Doulabi, Jenneke Klein-Nulend, Marco N. Helder

**Affiliations:** 1 Department of Biomedical Engineering, Faculty of New Sciences and Technologies, University of Tehran, Tehran, Iran;; 2 Department of Biomedical Engineering, Research Center for New Technologies in Life Science Engineering, University of Tehran, Tehran, Iran;; 3 School of Chemical Engineering, College of Engineering, University of Tehran, Tehran, Iran;; 4 Department of Oral and Maxillofacial Surgery/Oral Pathology, Amsterdam University Medical Centers-location VUmc and Academic Centre for Dentistry Amsterdam (ACTA), Amsterdam Movement Sciences, Amsterdam, the Netherlands;; 5 Department of Oral Cell Biology, Academic Centre for Dentistry Amsterdam (ACTA)-University of Amsterdam and Vrije Universiteit Amsterdam, Amsterdam Movement Sciences, Amsterdam, the Netherlands

**Keywords:** Alkaline phosphatase, β-tricalcium phosphate, Poly(lactic-co-glycolic) acid copolymer

## Abstract

**Background:**

One of the main challenges with conventional scaffold fabrication methods is the inability to control scaffold architecture. Recently, scaffolds with controlled shape and architecture have been fabricated using 3D-printing. Herein, we aimed to determine whether the much tighter control of microstructure of 3DP PLGA/β-TCP scaffolds is more effective in promoting osteogenesis than porous scaffolds produced by solvent casting/porogen leaching.

**Methods:**

Physical and mechanical properties of porous and 3DP scaffolds were studied. The response of pre-osteoblasts to the scaffolds was analyzed after 14 days.

**Results:**

The 3DP scaffolds had a smoother surface (R_a_: 22 ± 3 µm) relative to the highly rough surface of porous scaffolds (R_a_: 110 ± 15 µm). Water contact angle was 112 ± 4° on porous and 76 ± 6° on 3DP scaffolds. Porous and 3DP scaffolds had the pore size of 408 ± 90 and 315 ± 17 µm and porosity of 85 ± 5% and 39 ± 7%, respectively. Compressive strength of 3DP scaffolds (4.0 ± 0.3 MPa) was higher than porous scaffolds (1.7 ± 0.2 MPa). Collagenous matrix deposition was similar on both scaffolds. Cells proliferated from day 1 to day 14 by fourfold in porous and by 3.8-fold in 3DP scaffolds. ALP activity was 21-fold higher in 3DP scaffolds than porous scaffolds.

**Conclusion:**

The 3DP scaffolds show enhanced mechanical properties and ALP activity compared to porous scaffolds *in vitro*, suggesting that 3DP PLGA/β-TCP scaffolds are possibly more favorable for bone formation.

## INTRODUCTION

Bone is a tissue with self-repairing capacity; however, critical size bone defects (larger than 1 cm) usually cannot be regenerated by the inherent bone healing capacity. Treatment of such bone defects by autografts and allografts possesses several drawbacks, including inadequate availability, possibility of damage to the bone harvest site, and rejection^[^^1^^]^. Bone tissue engineering is a developing field aiming to tackle the problems with the current treatments of bone diseases. 

 Bioresorbable scaffolds are a key component in current bone tissue engineering approaches and provide a temporary mechanical and structural osteoconductive support. Polymers such as poly(ɛ-caprolactone), polylactic acid, and PLGA, belonging to poly(α-hydroxy esters) family, are FDA approved biodegradable materials broadly utilized to construct scaffolds for bone regeneration^[^^2^^]^. These polymers have adjustable degradation rates with degradation products that can be removed by natural pathways in the body^[^^2^^,^^3^^]^. However, poly(α-hydroxy esters) lack osteoconductivity and cell recognition sites^[^^2^^,^^4^^]^. Therefore, these polymers are often used as composites with osteoconductive materials, including biphasic calcium phosphate and β-TCP^[^^5^^-^^8^^]^.

 The 3D porous scaffolds fabricated by traditional approaches aim to mimic the structure of cancellous bone^[^^9^^]^. However, control over the internal structure with high accuracy is difficult^[^^10^^]^. Moreover, creating patient-specific scaffolds with shapes fitting the defect geometry is virtually impossible. To address these issues, 3D printing technology is currently extensively being used^[^^11^^]^. In this technology, the shape of the defect site is first identified using computed tomography or magnetic resonance imaging. Geometrical modelling is then conducted to generate a 3D computer-aided design model. The 3D scaffold is eventually printed layer-by-layer according to the designed model^[^^12^^]^.

 The current study attempted to determine whether the much tighter control of shape and nano/ microstructure of 3DP PLGA/β-TCP scaffolds is more effective in promoting osteogenesis than the 3D porous scaffolds produced by solvent casting/porogen leaching method, using the same components. This concept was evaluated by the detailed characterizations of scaffold properties (i.e. surface morphology and hydrophilicity, β-TCP particle dispersion, pore size, porosity, and compressive strength), as well as by the multi-parameter assessment of MC3T3-E1 pre-osteoblasts responses. These findings could provide solid insights in which the type of scaffold is preferred for future use in engineered bone applications.

## MATERIALS AND METHODS

**Porous scaffold fabrication by solvent casting/porogen leaching**

Scaffolds were fabricated using a previously described method^[^^13^^]^ with slight modifications. Briefly, sugar crystals (300-500 µm) were used as the porogen. A 10-mm diameter cylindrical glass mold filled with the sugar crystals was placed in a humidified sealed container for 16 h to partly fuse the crystals. PLGA (75/25, Purac Biomaterials, the Netherlands) was dissolved in DMSO (12.5% [w/w]; MP Biomedicals, France) and thoroughly mixed with β-TCP powder (NikCeram Razi, Isfahan, Iran) in a 2:1 (w/w) ratio. This PLGA/β-TCP mixture was then added dropwise to the sugar mold, allowing diffusion throughout the fused sugar crystals and transferred to -20 °C for 2 h to set the scaffold. The sugar crystals were subsequently leached out of the precipitated PLGA/β-TCP mixture in deionized water at room temperature for three days (three refreshments per day). All scaffolds were sterilized by UV for 30 min, followed by 1-h immersion in 70% ethanol. Scaffolds fabricated by this method are referred to as “porous” scaffolds.

**3DP scaffold fabrication**

PLGA granules were melted at 150 °C. β-TCP powder was subsequently mixed with the molten PLGA in a 2:1 ratio. The PLGA/β-TCP mixture was transferred to the heating tank of the 3D-bioprinter (RegenHU, Villaz-St-Pierre, Switzerland), and scaffolds were printed layer-by-layer at 125 °C and 29 revs/min. The inner diameter of the extruding needle was 300 µm. Cylindrical scaffolds with a diameter of 10 mm and height of 6 mm (total volume 0.471 cm^3^) were produced. 

**Characterization of scaffolds**

SEM (Zeiss EVO LS-15, Oberkochen, Germany) was used to study the surface morphology and dispersion of β-TCP particles, as well as the pore size of porous and 3DP scaffolds. Scaffolds were gold-coated (Edwards, Burgess Hill, UK) prior to imaging. The accelerating voltage of 10 kV was used for imaging. Pore size measurements (n = 5 per scaffold) were conducted randomly from SEM images. Surface hydrophilicity was evaluated by placing a drop of water on the surface of porous and 3DP scaffolds and by measuring the angle of water drop using a USB digital microscope (Veho, Hampshire, UK). Surface roughness was determined using a surface profilometer (Fanavari Kahroba Co., Tehran, Iran). For porosity measurement, scaffolds were immersed in ethanol under vacuum^[^^14^^]^. After 20 min, scaffolds were removed from ethanol, and the porosity was calculated using the following equation:

$p=\frac{\left(V_{1}-V_{3}\right)}{\left(V_{2}-V_{3}\right)}$

where *p* is scaffolds porosity, *V*_1_ is the initial volume of ethanol, *V*_2_ is the volume of ethanol containing the scaffold, and *V*_3_ is the volume of the remaining ethanol after scaffold removal.

An Instron 6022 universal testing machine (Instron Limited, High Wycombe, UK) with a load cell of 1 kN was used to determine the compressive strength of the scaffolds, which were then compressed at a rate of 1 mm/min. Values of load *F* (N) and time (s) were recorded during the test. The stress-strain curves were plotted, and compressive strength was calculated by 1% offset of the linear region. 

**Cell culture studies**

MC3T3-E1 pre-osteoblasts (ATCC, Manassas, VA, USA) were cultured in α-MEM (Life Technologies, Waltham, MA) containing 10% fetal bovine serum (BioWest SAS, Nuaille, France) and 1% penicillin-streptomycin-fungizone (Sigma-Aldrich®, St. Louis, MO, USA) in an incubator at 37 °C. Upon reaching desired confluency, cells were detached using 0.25% trypsin (Invitrogen, Waltham, MA, USA) and 0.1% EDTA (Merck, Darmstadt, Germany), re-suspended in osteogenic medium (α-MEM with 10% fetal bovine serum, 1% penicillin-streptomycin-fungizone, 50 µg/ml ascorbic acid, and 10 mM β-glycerophosphate) and seeded onto the porous and 3DP PLGA/β-TCP scaffolds at 5 × 10^5^ cells/cm^3^. Cell-seeded scaffolds were incubated at 37 °C up to 14 days. At day 14, scaffolds were removed from culture and cut into four equal parts. The parts were used to assess the cell morphology (part 1 construct), cell dispersion (part 2 construct), collagenous matrix deposition (part 3 construct), and ALP activity (part 4 construct), as described below.

**Cell seeding efficiency**

Cell seeding efficiency was measured 16 h after seeding the cells onto the scaffolds. Cell-containing scaffolds were transferred to a fresh plate and washed twice with PBS. AlamarBlue® solution (Invitrogen, Frederick, MD) was added to the scaffolds and incubated at 37 °C for 4 h. The solution was then collected from the scaffolds, and fluorescence was measured at 530 nm using a Synergy HT® spectrophotometer (BioTek Instruments Inc., Winooski, VT, USA). The number of cells attached to the scaffold (*N*_s_) was determined using a standard curve. The number of cells attached to the old plate (*N*_p_) was also determined following the same procedure. Seeding efficiency was calculated using the following equation^[^^15^^]^:

$\mathrm{Seeding efficiency}=\frac{N_{s}}{N_{s}+N_{p}}\times 100$

After performing the seeding efficiency test, scaffolds were returned to the incubator. Scaffolds were assayed in triplicate.

**Cell proliferation**

At days 3, 7, and 14 of cell culture in the scaffolds, cell-containing scaffolds were removed from culture, and the number of cells attached to the scaffolds was determined using AlamarBlue® assay. Cell proliferation was reported as the number of cells attached to the scaffolds at each time point divided by the number of cells attached to the scaffolds at day one. Three replicates were used for each scaffold type.

**Cell morphology and dispersion**

The morphology of the cells on porous and 3DP scaffolds was studied using SEM. At day 14, scaffolds were cut into four equal parts, and the first part was used for cell morphology analysis. Part 1 of the cell-containing scaffolds was fixed in glutaraldehyde (4% v/v), followed by dehydration in a series of ethanol solutions with increasing concentrations (50, 70, 80, 90, and 100%). After drying, the fixed samples were gold-coated and observed using a scanning electron microscope. To determine cell dispersion in porous and 3DP scaffolds, another part (part 2) of the cell-containing scaffolds was washed in PBS and fixed using formaldehyde (4% v/v). Cells in the fixed samples were fluorescently stained by using DAPI and visualized by an inverted microscope (Leica Microsystems, Wetzlar, Germany).

**Collagenous matrix deposition by cells**

In order to assess total collagen deposition by the pre-osteoblasts in the scaffolds, staining with picrosirius red (Chondrex Inc., Redmond, WA, USA) was performed. Part 3 of the cell-containing scaffolds were washed with PBS and fixed using formaldehyde (4% v/v). The fixed samples were stained with picrosirius red for 2 h. Samples were washed in acidified water (0.5% aqueous acetic acid) and imaged with a stereo microscope (Nikon, Tokyo, Japan). To quantify collagen deposition, the dye was extracted from the samples by immersion in 0.2 M of NaOH/methanol (1:1, v/v) solution for 30 min. The absorbance of the extracted solution was read at 490 nm with a microplate reader (BioRad Laboratories Inc., Veenendaal, the Netherlands)^[^^16^^]^. Measurements were normalized to the weight of the dried samples. We were unable to count the cells in the cell-scaffold construct (part 3) prior to staining to normalize collagen deposition to the cell numbers since lysing the cells for counting would have interfered with picrosirius red staining.

**ALP activity and total protein measurement**

As an indication of the osteogenic activity of the cells in porous and 3DP scaffolds, part 4 of the cell-containing scaffolds was used for ALP activity measurement. Samples were separately placed in falcon tubes, and CyQuant® lysis buffer (Invitrogen, Carlsbad, CA, USA) was added to the samples to lyse the cells. Samples were freezed, and three cycles of freeze-thawing was performed. ALP activity was determined using P-Nitrophenylphosphate (Merck) at pH 10.3 as the substrate^[^^17^^]^, and absorbance was read at 410 nm. To determine the total cellular protein, a BCA Protein Assay Kit (Pierce^TM^, Rockford, IL, USA) was used according to the manufacturer’s instructions. ALP activity was normalized to total protein and reported as nmol/µg cellular protein. For ALP activity staining, nitro blue tetrazolium/5-bromo-4-chloro-3-indolyl phosphate (Roche, Germany) was applied as per protocols provided by the manufacturer.

**Statistical analysis**

Data were reported as mean ± SD (n = 3) and compared using unpaired two-tailed t-test (GraphPad Software, La Jolla, CA, USA). A value of *p* < 0.05 was considered statistically significant.

## RESULTS

**Characterization of porous and 3DP PLGA/β-TCP scaffolds**

**Fig. 1:**
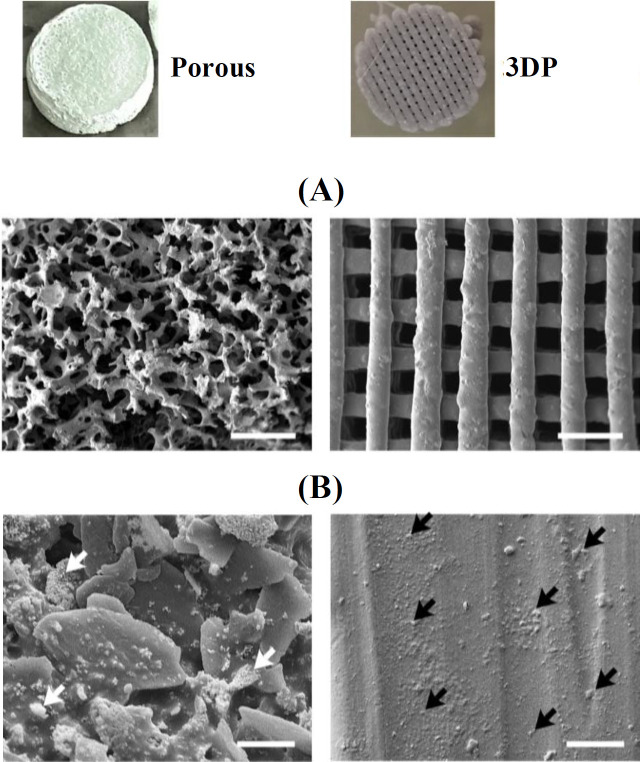
SEM images of porous and 3DP PLGA/β-TCP scaffolds. (A) Surface morphology of the scaffolds;  (B) distribution of β-TCP particles on the surface of scaffolds. White arrows, β-TCP aggregates on the porous scaffold;  black arrows, β-TCP particles on the 3DP scaffold. Scale bar, 10 µm

**Fig. 2:**
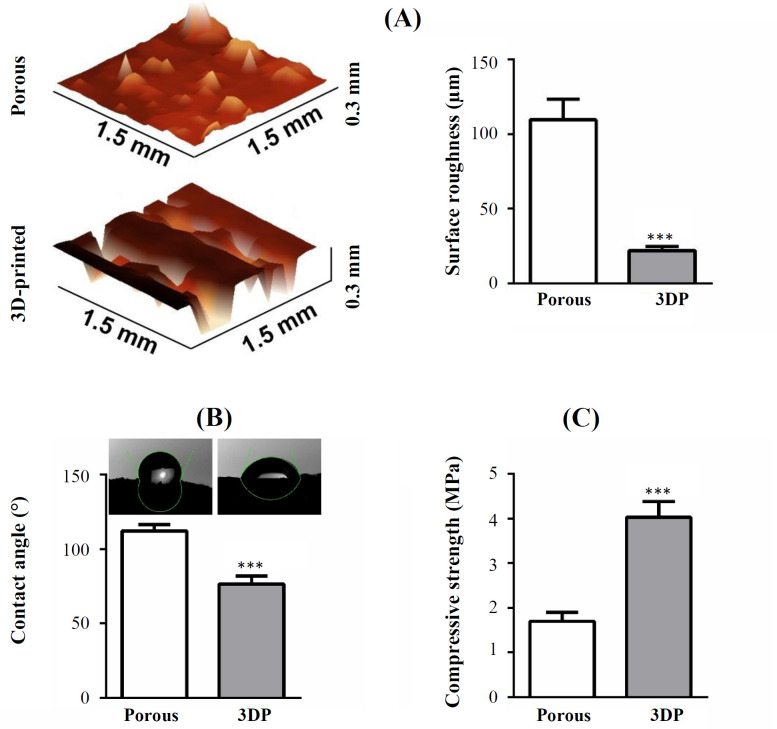
Physical properties of porous and 3DP PLGA/β-TCP scaffolds. (A) Surface roughness, (B) water contact angle, and  (C) compressive strength of the scaffolds. Data are mean ± SD (n = 3). ^***^Significantly different from porous scaffolds, *p* < 0.001

The porous scaffolds had a pore size of 408 ± 90 (mean ± SD) µm and a porosity of 85 ± 5%. The pore size of 3DP scaffolds was 315 ± 17 µm, and its porosity was 39 ± 7% (Fig. 1A). The distribution of β-TCP particles on the surface was more homogeneous for the 3DP scaffolds in comparison to the porous scaffolds (Fig. 1B). β-TCP aggregates were visible only on the porous scaffolds surface (Fig. 1B). The 3DP scaffolds had smoother (R_a_: 22 ± 3 µm, mean ± SD) and more regular surface compared with the highly rough surface of the porous scaffolds (R_a_: 110 ± 15 µm), which had an irregular structure (*p* = 0.0004, n = 3; Fig. 2A). The 3DP scaffolds had a more hydrophilic surface (water contact angle: 76 ± 6°) relative to the porous scaffolds (water contact angle: 112 ± 4°; *p* = 0.0009, n = 3; Fig. 2B). Compressive strength of the 3DP scaffolds (4.0 ± 0.3 MPa) was higher (*p* = 0.0006, n = 3) than the porous scaffolds (1.7 ± 0.2 MPa; Fig. 2C).

**Cell morphology and dispersion on porous and 3DP** **PLGA/β-TCP** **scaffolds**

**Fig.3:**
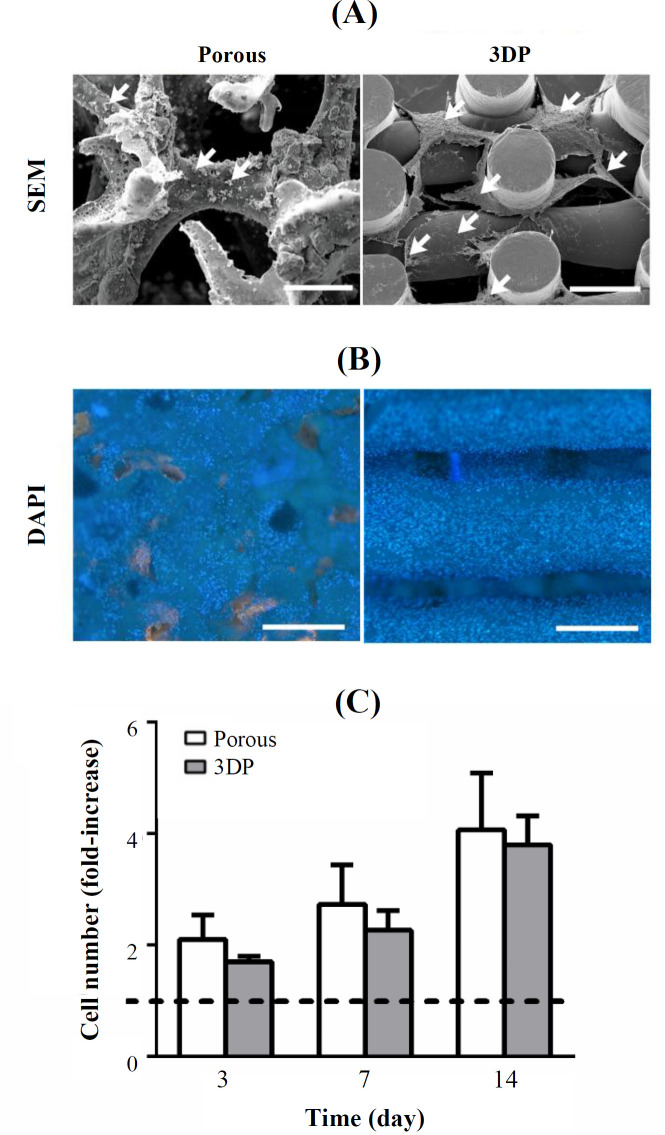
Interaction of MC3T3-E1 pre-osteoblasts with porous and 3DP PLGA/β-TCP scaffolds. (A) Morphology, (B) dispersion, and (C) proliferation of the cells on scaffolds. Scale bar, 500 µm. Data are mean ± SD (n = 3)

Cells dispersed on the surface of the porous scaffolds had less spread morphology than that of the 3DP scaffolds (Fig. 3A). A dense cellular network was formed on and between the struts of the 3DP scaffolds, and cellular bridging between adjacent struts was observed (Fig. 3A). MC3T3-E1 pre-osteoblasts dispersion on porous and 3DP scaffolds was visualized by DAPI staining after 14 days of culture (Fig. 3B). Slightly higher cell coverage was observed on the 3DP scaffolds compared with the porous scaffolds. 

**Cell seeding efficiency and proliferation on porous and 3DP** **PLGA/β-TCP** **scaffolds**

Cell seeding efficiency on the two scaffold types was measured 16 h after cell seeding onto the scaffolds. Seeding efficiency was 62 ± 3% (mean ± SD) for the porous and 65 ± 2% for the 3DP scaffolds. Difference in seeding efficiency was not statistically significant (*p* = 0.2230, n = 3). Cell proliferation on porous and 3DP scaffolds was analyzed at days 3, 7, and 14 relative to day 1 (Fig. 3C). Cells proliferated on the porous scaffolds by 2.1, 2.7, and 4.0-fold after 3, 7, and 14 days of culture, respectively. Cells proliferated on the 3DP scaffolds by 1.7, 2.3, and 3.8-fold after 3, 7, and 14 days of culture, respectively. The differences in cell proliferation between the two scaffold types was statistically insignificant (*p* = 0.0234, n = 3).

**Collagenous matrix deposition by cells on porous and 3DP** **PLGA/β-TCP** **scaffolds**

**Fig. 4:**
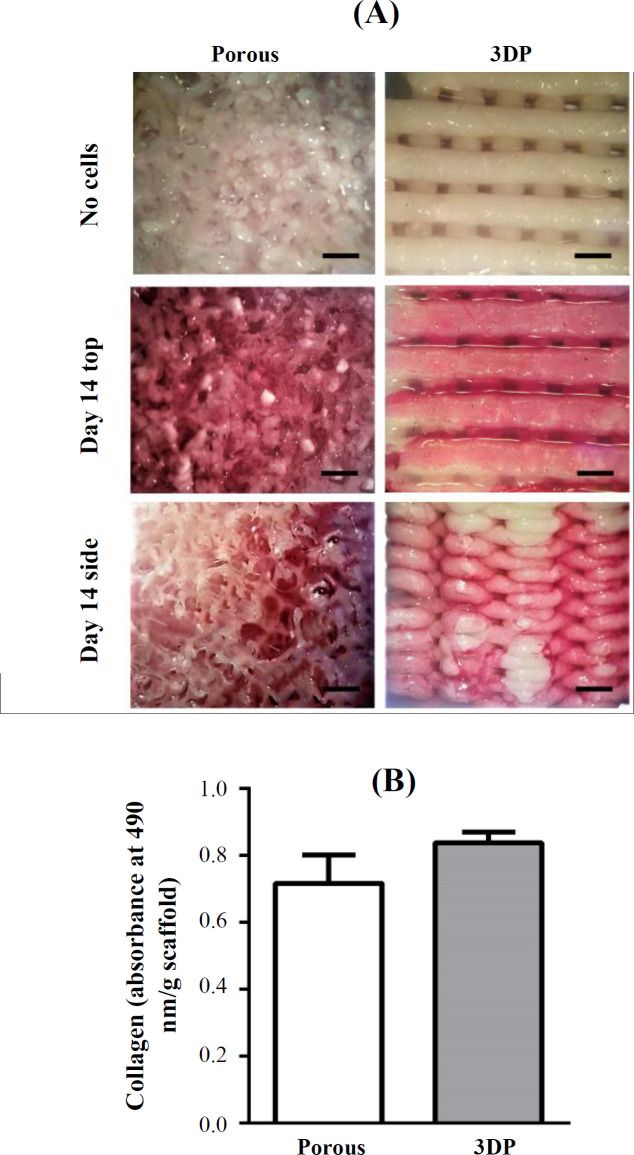
Collagen deposition by MC3T3-E1 pre-osteoblasts on porous and 3DP scaffolds at 14 days of culture. (A) Collagen-stained scaffolds; (B) quantification of collagen deposition. Scale bar, 500 µm. Data are mean ± SD (n = 3)

Collagen deposition by the cells on the two scaffold types was assessed using picrosirius red staining. At day 0 (prior to cell seeding), scaffolds stained with picrosirius red did not display any red color (Fig. 4A). At day 14, collagen deposition stained in red was noticed in both the top view and the side view of porous and 3DP scaffolds (Fig. 4A). Collagen deposition was not significantly different (*p* = 0.0817, n = 3) between the two scaffolds (Fig. 4B).

**ALP activity by cells on** **porous and 3DP PLGA/β-TCP scaffolds**

**Fig. 5:**
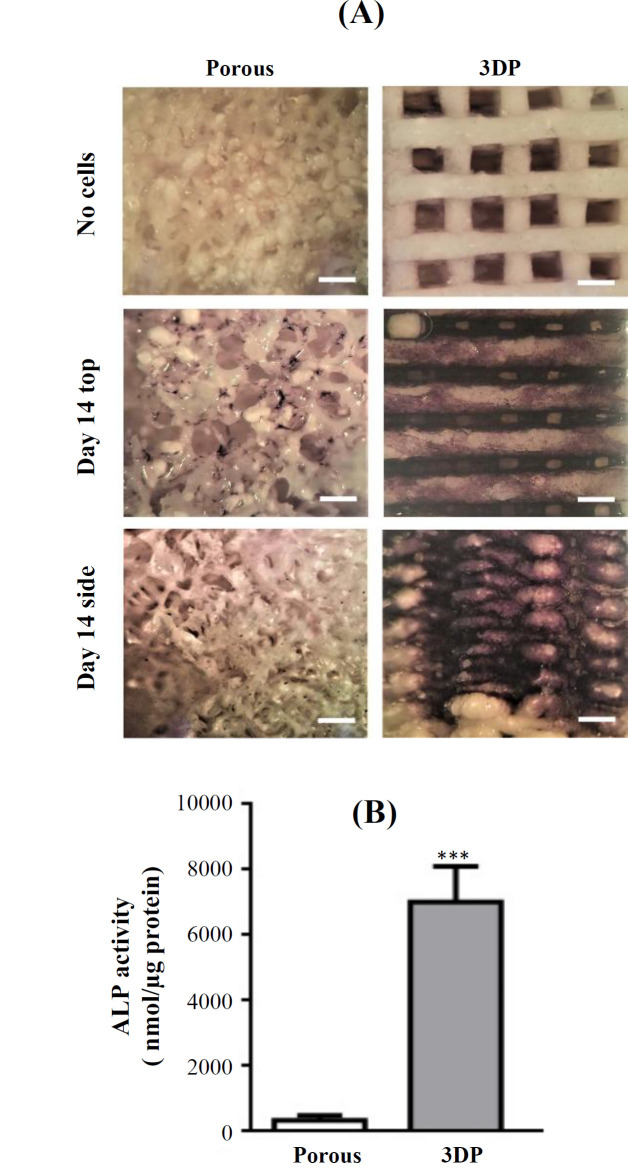
ALP activity of MC3T3-E1 pre-osteoblasts on porous and 3DP scaffolds after 14 days of culture. (A) ALP-stained scaffolds. (B) Quantitative analysis of ALP activity on the scaffolds. Scale bar, 500 µm. Data are mean ± SD (n = 3). ^***^Significantly different from porous scaffolds, *p* < 0.001

ALP activity by the cells on the two scaffold types was visualized and measured after 14 days of culture. Cells were stained for ALP activity using NBT/BCIP. At day 0 (prior to cell seeding), scaffolds stained with NBT/BCIP did not display any purple color (Fig. 5A. After 14 days of culture, ALP activity (purple) was observed in the top view and the side view of the 3DP scaffolds. Scarce ALP activity was observed on the porous scaffolds (Fig. 5A). ALP activity was also quantitatively measured and was significantly higher by 21-fold (*p* = 0.0002, n = 3) on the 3DP scaffolds compared with the porous scaffolds (Fig. 5B).

## DISCUSSION

It is generally accepted that 3D structure and internal architecture of bone tissue engineering scaffolds play pivotal roles in the successful functioning of the scaffold. Parameters such as scaffold pore size, porosity, and 3D structure greatly influence the formation of new tissue^[^^18^^,^^19^^]^. In this study, we assessed the scaffold characteristics and the response of MC3T3-E1 pre-osteoblasts on porous versus 3DP PLGA/β-TCP scaffolds. We found that in comparison to the porous scaffolds: (i) the 3DP scaffolds had a relatively smoother surface with a more regular structure; (ii) the dispersion of β-TCP particles was more homogeneous on the surface of the 3DP scaffolds; (iii) the surface of the 3DP scaffolds was more hydrophilic; (iv) compressive strength of the 3DP scaffolds was significantly higher; (v) a more dense cellular network was formed on and between the struts of the 3DP scaffolds; (vi) seeding efficiency and cell proliferation were similar; (vii) cells deposited comparable amounts of collagenous matrix; (viii) ALP activity was significantly higher on the 3DP scaffolds. 

 In this study, porous and 3DP scaffolds were fabricated from the same PLGA/β-TCP material. Therefore, the main parameters potentially influencing cellular responses were the architecture, physical and mechanical characteristics of the scaffolds. Internal architecture of scaffolds is identified by several interdependent parameters such as pore size, porosity, and strut diameter so that it proved difficult to attribute a particular cellular response to a certain parameter. For instance, it has been shown that cells can sense and respond to structural parameters such as the orientation of scaffolds struts, fibers, and pores^[^^20^^-^^22^^]^. In our study, the porous scaffolds had a random internal orientation, whereas the struts of the 3DP scaffolds had regular parallel orientation in each layer. Therefore, cells attached to the struts of the 3DP scaffolds were provided with a relatively regular surface without deep hills and valleys allowing them to stretch and form cellular networks, while the highly random internal orientation and topography of the porous scaffolds prevented the cells from forming such dense cellular networks (Fig. 3A).

 Scaffold surface roughness is another important physical factor influencing cellular functions, especially osteogenesis^[^^23^^,^^24^^]^. This feature may occur through topography-induced modulation of the cell mechanotransduction^[^^25^^]^. Highly rough surfaces (high *R*_a_ values) as well as smooth surfaces (low *R*_a_ values) are not desirable for cellular functioning^[^^26^^,^^27^^]^. For example, surfaces with micrometer-sized roughness may prevent cells to effectively interact with other cells as it is difficult to cross over the surface irregularities^[^^28^^]^. Moreover, on more hydrophilic surfaces, cells generally show good proliferation and differentiation, which has also been indicated for the currently used pre-osteoblastic cell line MC3T3-E1 by Wei *et al.*^[^^29^^]^, whose findings appear to be confirmed by our results. From the ALP activity data, we can deduce that the R_a_ value of the porous scaffolds was too high to optimally promote ALP activity as an osteogenic marker, though the slightly rough surface of the 3DP PLGA/β-TCP scaffolds facilitated osteoblast maturation potential of the MC3T3-E1 pre-osteoblasts upon the application of osteogenic media. We also concluded that the 3DP PLGA/β-TCP scaffolds having a hydrophilic surface with slight roughness resulted from β-TCP particles might be favorable for pre-osteoblast osteogenic activity.

 Another pivotal aspect of the scaffolds is their pore structure. There are conflicting reports in the literature on the impact of porosity and pore size on cellular responses. In one research, it has been displayed that apatite/collagen composite scaffolds containing pores with dimensions of 50-300 µm and porosities of 49-79% result in insignificant difference in MC3T3-E1 pre-osteoblast proliferation^[^^30^^]^. In another study, it has been described that mesenchymal stem cells seeded on hydroxyapatite scaffolds in dynamic culture have higher proliferation in scaffolds with 500 µm pores than those of 200-µm pores but have higher osteogenic differentiation in the 200 µm pore scaffolds^[^^31^^]^. The effect of the porosity of β-TCP scaffolds on osteogenic differentiation of *mesenchymal stem* cells was investigated in another study^[^^32^^]^. It has been evidenced that porosity does not influence the osteogenic differentiation of *mesenchymal stem*  cells *in vitro*, while scaffolds with porosity of 65% result in higher ALP activity *in vivo *compared with 25% and 75% porosity scaffolds^[^^32^^]^. In our study, the porous scaffolds had the pore sizes of ~ 400 µm and a porosity of 85%, while the 3DP scaffolds had pore sizes of ~315 µm and a porosity of 39%. MC3T3-E1 pre-osteoblasts proliferation was similar on porous and 3DP scaffolds, while significantly higher ALP activity was obtained on the 3DP scaffolds compared to the porous scaffolds. Our results are in agreement with the aforementioned data in the literature, showing that the higher pore size and porosity do not necessarily lead to higher ALP activity.

Calcium phosphates, including β-TCP and hydroxyapatite, are well known for their osteo-conductive properties^[^^33^^]^. Some reports have also claimed osteoinductivity for these materials^[^^34^^,^^35^^]^. In this study, the distribution of β-TCP particles on the surface of the scaffolds is likely to influence the ALP activity of the pre-osteoblasts. β-TCP particles were more homogeneously dispersed on the 3DP scaffolds surface than that of the porous scaffolds. Moreover, aggregations of β-TCP were visible on the porous scaffolds surface, which is unfavorable for homogeneous osteogenesis by pre-osteoblasts. The inhomogeneous dispersion of β-TCP particles in the porous scaffolds was due to low viscosity of the PLGA/DMSO solution, causing premature gravity precipitation during the solidification step after casting the mixture over the fused sugar mold. On the other hand, the molten PLGA/β-TCP mixture used for 3D-printing was far more viscous, thus allowing a more homogeneous distribution of β-TCP particles in the PLGA matrix after 3D-printing. This property represents an important advantage of the 3DP scaffolds in this study. We speculate that the more homogeneous distribution of β-TCP particles and proper surface roughness in the 3DP scaffold may have had greater impact on cellular responses, especially ALP activity, as opposed to other physical factors such as pore size and porosity.

 Last but not least, it has been shown that the mechanical properties of bone tissue engineering scaffolds can modulate cellular responses (i.e. cell morphology, proliferation, migration, and differentiation)^[^^36^^,^^37^^]^. The compressive strength of the 3DP PLGA/β-TCP scaffolds was significantly higher than the porous scaffolds. This attribute was expected based on the lower porosity of the 3DP scaffolds (39 ± 7%) compared with the porous scaffolds (85 ± 5%). It has previously been suggested that the compressive strength of scaffolds decreases significantly with increasing scaffolds porosity^[^^38^^]^. Higher compressive strength of the 3DP PLGA/β-TCP scaffolds indicates another advantage of these scaffolds compared with porous scaffolds for bone tissue engineering.

 Cell seeding efficiency of porous and 3DP PLGA/β-TCP scaffolds were similar after 16 h of culture. Moreover, cells had a similar proliferation rate on the two scaffold types. Besides, collagenous matrix deposition was not significantly different between the two scaffolds. This issue indicates that although the difference in the surface structure and internal architecture between the two scaffold types resulted in different cell attachment and spreading pattern, it did not have a significant effect on the proliferation and matrix deposition ability of the cells. However, it did have a significant effect on ALP activity of pre-osteoblasts on the 3DP scaffolds compared with the porous scaffolds. In addition to the homogeneous distribution of β-TCP particles on the 3DP scaffolds discussed earlier, higher ALP activity on the 3DP scaffolds can be attributed to more effective cell-cell contacts on these scaffolds compared with cell-cell contacts on the porous scaffolds. Cell-cell contacts have been demonstrated to be essential for inducing osteogenic differentiation. Upon cell-cell contact, cells can exchange bioactive molecules through gap junctions to enable differentiation^[^^39^^,^^40^^]^. Therefore, suitable surface roughness, homogeneous dispersion of β-TCP particles on the surface, and effective cell-cell contact on the 3DP scaffolds led to the higher ALP activity as an osteogenic marker by pre-osteoblasts in these scaffolds, as compared with the porous scaffolds fabricated by solvent casting/porogen leaching. These findings have provided further knowledge on the response of pre-osteoblasts to ordered 3DP or random porous scaffolds fabricated from the same PLGA/β-TCP material, which may have important implications for fine-tuning scaffold designs for bone tissue engineering.

Proliferation, matrix deposition, and ALP activity as an osteogenic marker of MC3T3-E1 pre-osteoblasts in porous or 3DP PLGA/β-TCP scaffolds were evaluated in this study. Our data indicate that porous and 3DP scaffolds equally support the pre-osteoblasts proliferation and matrix deposition, though only the 3DP scaffolds showed enhanced mechanical properties and ALP activity *in vitro. *This observation suggests that the 3DP PLGA/β-TCP scaffolds may be more favorable for *in vivo* bone formation than the porous PLGA/β-TCP scaffolds.
